# Robust Video Stabilization Using Particle Keypoint Update and *l*_1_-Optimized Camera Path

**DOI:** 10.3390/s17020337

**Published:** 2017-02-10

**Authors:** Semi Jeon, Inhye Yoon, Jinbeum Jang, Seungji Yang, Jisung Kim, Joonki Paik

**Affiliations:** 1 Department of Image, Chung-Ang University, 84 Heukseok-ro, Dongjak-gu, Seoul 06974, Korea; semi2530@gmail.com (S.J.); inhyey@gmail.com (I.Y.); jinbeum23@gmail.com (J.J.); 2 ADAS Camera Team, LG Electronics, 322 Gyeongmyeong-daero, Seo-gu, Incheon 22744, Korea; 3 Future Technology R&D, SK Telecom, Sunae-dong, Bundang-gu, Seongnam 13595, Korea; yangs@sk.com (S.Y.); joyful.kim@sk.com (J.K.)

**Keywords:** video stabilization, feature extraction, camera motion estimation, video enhancement

## Abstract

Acquisition of stabilized video is an important issue for various type of digital cameras. This paper presents an adaptive camera path estimation method using robust feature detection to remove shaky artifacts in a video. The proposed algorithm consists of three steps: (i) robust feature detection using particle keypoints between adjacent frames; (ii) camera path estimation and smoothing; and (iii) rendering to reconstruct a stabilized video. As a result, the proposed algorithm can estimate the optimal homography by redefining important feature points in the flat region using particle keypoints. In addition, stabilized frames with less holes can be generated from the optimal, adaptive camera path that minimizes a temporal total variation (TV). The proposed video stabilization method is suitable for enhancing the visual quality for various portable cameras and can be applied to robot vision, driving assistant systems, and visual surveillance systems.

## 1. Introduction

The demand for a compact, portable camera is rapidly growing because of popularized consumer hand-held cameras with easy handling and compact size such as mobile cameras, digital cameras, digital camcorders, drone cameras, and wearable cameras. With the advancement of cloud services, acquisition of high quality videos becomes more important to share contents without the barriers of time and space. However, video sequences are subject to undesired vibrations due to camera shaking caused by poor handling and/or a dynamic, unstable environment. To overcome this problem, various video stabilization methods have been developed to improve the visual quality of various hand-held cameras [1]. A mechanical video stabilization system controls the camera vibrations using the gyro sensor or accelerometer. It either moves the lens to change the light path and the optical axis or uses an internal sensor to minimize the shaky motion. In spite of the high performance, the mechanical and optical video stabilizer is not suitable for portable camera because of the increased volume and cost of the system. On the other hand, an image processing-based video stabilizer can efficiently remove the movement of video frames without extra cost of additional hardware devices.

An image processing-based video stabilization method generally consists of two steps: (i) removing undesired motion by smoothing the camera path and (ii) rendering the stabilized frames [2]. Existing video stabilization systems can be classified by the camera path estimation method. Early two-dimensional (2D) stabilization methods used the block matching algorithm to estimate inter-frame motion vectors. Jang et al. estimated the optimal affine model between adjacent frames by using a variable block size [3]. Xu et al. proposed a video stabilization algorithm using circular block matching and least square fitting [4]. Since the 2D block matching-based methods can easily estimate the camera path, they are applied in various applications [5]. However, they are sensitive to noise and produce a matching error between acquired video frames under a dynamic environment. An improved 2D video stabilization method used the optical flow to estimate the global camera path. Chang et al. used the Lucas-Kanade optical flow estimation algorithm to define an affine motion model between frames, and stabilized the camera path by motion compensation [6]. Matsushita et al. estimated the camera path using the homography between adjacent frames and smoothed the global path using a Gaussian kernel [7]. Xu et al. used Horn-Schunck optical flow estimation algorithm to compute an affine model between successive frames and smoothed camera path by model-fitting filter [8]. Although optical flow-based stabilization methods can compute an affine motion model in a simple, flexible manner, they fail to stabilize multiple objects with different distances at the same time. To improve the quality of stabilized video, an alternative approach used feature points to estimate a rotation- and scale-invariant camera path. Battiato et al. used the scale invariant feature transform (SIFT) to estimate the camera path and reduce the estimation error using the least squares algorithm [9]. Lee et al. used trajectories of SIFT feature points to estimate the camera path and minimized an energy function to smooth the camera path with reducing geometric distortion [10]. Xu et al. estimated motion parameters of the affine model using the fast accelerated segment test (FAST) algorithm for video stabilization [11]. Nejadasl et al. stabilized calibrated image sequence using the Kanade-Lucas-Tomasi (KLT) tracker and SIFT [12]. Cheng et al. presented motion detection using the speeded up robust features (SURF) and modified random sample consensus (RANSAC) for video stabilization [13]. To define a more powerful 2D camera model, the locally estimated camera path are proposed. Liu et al. modeled mesh-based 2D camera motion with bundled camera path to improve the video stabilization performance [14], and Kim et al. classified background feature points using the KLT tracker [15]. Although 2D video stabilization methods are faster and robust because of the use of a linear transformation, they fail to estimate the optimal camera path in textureless regions.

Currently, 3D camera motions are estimated based on the image segmentation result to improve the quality of a video. Liu et al. proposed a 3D video stabilization method using structure from motion and spatial warping to preserve 3D structures [16]. Zhou et al. generated labeled frames using 3D point cloud and estimated the homography of each label to reduce distortion in textureless regions [17]. The 3D stabilization methods can generate higher quality results and are suitable for an accurate video analysis [18,19]. However it is hard to implementation in real-time or near real-time service because of the high computational complexity, and these methods have the common problem of the parallax caused by feature tracking failure in flat region.

To solve these problems, this paper presents a novel video stabilization algorithm using a robust feature detection method to improve existing 2D methods instead of the less robust 3D methods. The proposed algorithm redefines important feature points using particle keypoints. The homography is accurately estimated by detecting robust particle keypoints. Undesired motions are removed by minimizing the temporal total-variation of the camera path. As a result, the proposed method provides a significantly increased visual quality of shaky video acquired by a handheld camera.

This paper is organized as follows. Section 2 presents theoretical background of video stabilization. Section 3 presents the robust feature extraction and matching based video stabilization and Section 4 presents the optimal camera path estimation. Experimental results are given in Section 5, and Section 6 concludes the paper.

## 2. Theoretical Background

**Figure 1:**
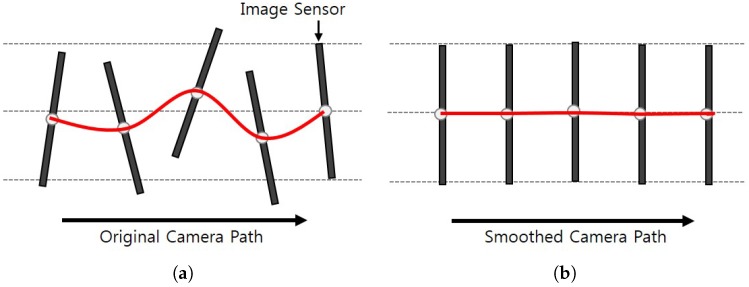
Video acquisition process using a complementary metal oxide semiconductor (CMOS) sensor portable camera: (**a**) input shaky camera path and (**b**) the smoothed camera path.

Digital video stabilization plays an important role of a stabilized sensor in acquiring high-quality video with preserving information for visual perception. A portable or wearable camera produces jitter and an undesired camera path because of various unstable video acquisition environments with camera shaking. Specifically, we can observe the geometric distortion of the video due to the mislocation of the pixels as shown in Figure 1a. The camera path is not consistent with camera coordinate system from the world coordinate system’s point of view. Since a perspective distortion is generated by undesired camera motion and rotation, the geometric transformation in the sensor output generates unstable video frames. For that reason, the proposed video stabilization algorithm compensates the perspective distortion caused by the transformation of the acquired video as shown in Figure 1b.

The shaky video can be considered as a geometrically transformed version of the ideally stable video. The relationship between feature points in the original and the shaky frames is defined in the homogeneous coordinate as

(1) q=Hp,

where H represents the homography, $p={\left[x,y,1\right]}^{T}$ a feature point in the original frame, and $q={\left[\hat{x},\hat{y},1\right]}^{T}$ its correspondence point in the shaky fame. The homography is generally estimated using the correspondences between adjacent frames. Although state-of-the-art feature extraction algorithms can detect distinguishable keypoints regardless of scale change, rotation, and brightness change, these methods fail to estimate the accurate homography of the images including a large flat region without any salient texture. The incorrectly estimated homography significantly degrades the performance of the video stabilization with an erroneous camera path.

To solve these problems, we extract robust feature points to estimate the optimal homography of the textureless region. By updating important feature points in flat regions using the particle keypoint, the proposed method can significantly remove undesirable jitter using the optimally estimated homography in the entire image. The proposed method can also improve the visual quality without expensive optical devices by reconstructing stable video with a significantly reduced perspective distortion.

## 3. Feature Extraction and Matching for Robust Video Stabilization

**Figure 2:**
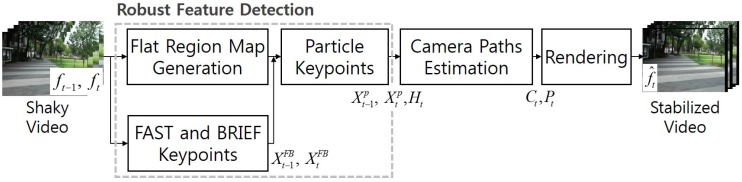
Block diagram of the proposed video stabilization method.

The proposed video stabilization method estimates the optimal camera path of a certain length of video by redefining robust feature points and it is an extended version of Jeon’s work [20]. Figure 2 shows the block diagram of the proposed video stabilization method. The proposed algorithm consists of three steps: i) robust feature detection, ii) estimation of camera path, and iii) rendering to reconstruct a stabilized video.

Given a pair of input shaky video frames $f_{t-1}$ and $f_{t}$, the flat region map is generated. The FAST and BRIEF keypoints $X_{t-1}^{FB}$, $X_{t}^{FB}$ are extracted in $f_{t-1}$ and $f_{t}$, respectively. The particle keypoints in the two frames, $X_{t-1}^{P}$ and $X_{t}^{P}$, are generated using statistical analysis of extracted FAST and BRIEF keypoints in flat regions. After that, the global camera path $C_{t}$ is estimated by the optimal homography $H_{t}$, and the smoothed camera path $P_{t}$ is then estimated using a variational method. As a result, the stabilized frame ${\hat{f}}_{t}$ is obtained using the estimated camera path.

### 3.1. Flat Region Map Generation for Feature Extraction

Conventional video stabilization methods enhance the quality of a consumer video by estimating and smoothing the global path. Existing video stabilization methods assumed that temporally adjacent frames are related by a homography, which is robust to camera transformation, and the global camera path can be easily estimated using the geometric transformation. The global camera path is estimated by matching feature points that are robust to a geometric transformation. However, existing methods fail to detect feature points in a flat region. In addition, an inaccurately estimated homography in a textureless region further degrades the stabilization performance. In order to solve this problem, the proposed method generates the flat region map and the optimal camera path by redefining important keypoints in a flat region.

**Figure 3:**
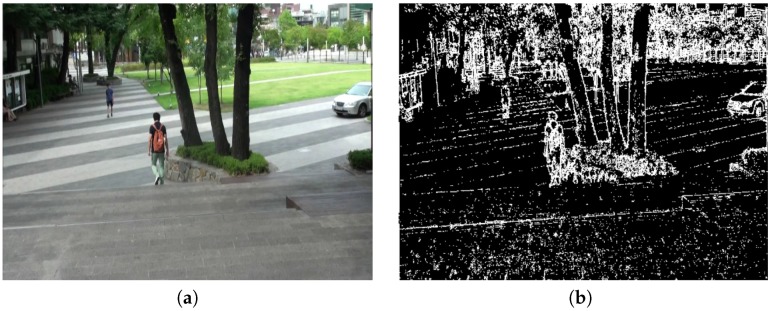
Example of a flat region map: (**a**) an input image and (**b**) its flat region map using the proposed method.

A textureless region is extracted using the flat region map. A spatially smoothed frames are obtained by convolving the shaky frames $f_{t-1}$ and $f_{t}$ with a 3×3 Gaussian low-pass filter. The frames are divided into flat and active regions using the absolute difference of the original frame and its smoothed version. As a result, the estimated flat region map is used to redefine robust feature points. Figure 3 shows the *t*-th original shaky frame and the corresponding flat region map.

### 3.2. Robust Feature Matching between Adjacent Frames

Matching of features between temporally adjacent frames is very important to understand the geometric relationship of two frames and detect specific objects in video [11,21]. Various feature detection methods were proposed and widely applied to detect a common region in two images [22]. Harris et al. proposed a seminal model to detect corner points where shifting a local window in any directions yields a large change in appearance [23]. Lowe proposed the scale-invariant feature transform (SIFT) that generates an image pyramid using the difference of Gaussian (DoG), and then keypoints are detected at the local maxima in the image pyramid [24]. Although SIFT can detect scale- and rotation- invariant feature points, the computational complexity is a bottleneck of video applications. To solve this problem, Bay et al. proposed the speeded up robust features (SURF) that uses an approximated filters and integral images to reduce the processing time [25]. Recently, a number of intensity-based feature point detection algorithms were proposed. Rosten et al. proposed a faster corner detection algorithm using an accelerated segment test, which is called FAST [26]. Calonder et al. proposed a simple description method using the binary robust independent elementary features (BRIEF), which compares image intensities of sampling pairs [27]. More binary descriptors were proposed using a special sampling pattern to compensate the orientation of keypoints [28,29].

The proposed method combines FAST and BRIEF for fast, accurate extraction of feature points. FAST extracts feature points by comparing intensities with 16 neighborhood pixels in the circle. We determine the corner if the intensity of the *n* contiguous neighborhood pixels $I_{p\to x}$ are all brighter than that of the candidate pixel $I_{p}$, or if they are all darker than that of the candidate pixel $I_{p}$. To arrange the neighborhood pixels in order of the amount of information about whether the candidate pixel *p* is a corner, the decision tree classifier is trained using the iterative Dichotomiser 3 (ID3) algorithm. The keypoints *p* is defined as

(2) $$S_{p\to x}=\left\{\begin{array}{ccc} d, & I_{p\to x}\leq I_{p}-t & \left(darker\right)\\ s, & I_{p}-t<I_{p\to x}<I_{p}+t & \left(similar\right)\\ b, & I_{p}+t\leq I_{p\to x} & \left(brighter\right) \end{array},\right.$$

where *x* represents the neighborhood that is selected by decision tree using the ID3 algorithm, and *t* the threshold for comparing intensity. We used *t*=0.2 for the experimentally best result. BRIEF identifies local feature points by comparing intensities of sampling pairs. The homography can be computed very efficiently because a binary string can be matched using the hamming distance by the XOR operation.

The FAST keypoints are extracted between two adjacent video frames $f_{t-1}$ and $f_{t}$ to determine the distribution of random particle keypoints. The descriptors are generated using BRIEF and matched using the hamming distance. The extracted FAST and BRIEF keypoints are denoted as $X_{t-1}^{FB}=\left\{\left(x_{t-1}^{1},y_{t-1}^{1}\right),\cdots ,\left(x_{t-1}^{M},y_{t-1}^{M}\right)\right\}$ and $X_{t}^{FB}=\left\{\left(x_{t}^{1},y_{t}^{1}\right),\cdots ,\left(x_{t}^{M},y_{t}^{M}\right)\right\}$. Next, particle keypoints are randomly generated in a flat region to detect robust feature points. The distribution of *N* particle keypoints $X_{t-1}^{P}=\left\{\left(x_{t-1}^{1},y_{t-1}^{1}\right),\cdots ,\left(x_{t-1}^{N},y_{t-1}^{N}\right)\right\}$ and $X_{t}^{P}=\left\{\left(x_{t}^{1},y_{t}^{1}\right),\cdots ,\left(x_{t}^{N},y_{t}^{N}\right)\right\}$ are characterized as Gaussian functions $G({\bar{X}}_{t-1}^{FB},\sum_{t-1})$ and $G({\bar{X}}_{t}^{FB},\sum_{t})$ in flat regions of frames $f_{t-1}$ and $f_{t}$, respectively. The Gaussian distribution is given as

(3) $$G\left(\mu ,\sigma^{2}\right)=\frac{1}{\sqrt{2\pi \sigma^{2}}}e^{\frac{-{\left(x-\mu \right)}^{2}}{2\sigma^{2}}},$$

where *μ* and *σ* respectively represent the mean and standard deviation of the distribution. The descriptor matches the frames in the sense of the distance between particle keypoints and FAST and BRIEF keypoints. The descriptor $D_{t}$ of *t*-th frame is defined as

(4) $$D_{t}=X_{t}^{P}-X_{t}^{FB}.$$

Final correspondences are matched using the sum of squared difference (SSD) of the descriptors of two frames. The descriptor is used to match robust keypoints in the flat region using particle keypoints. Finally, the optimal homography $H_{t}$ is estimated using random sample consensus (RANSAC) to eliminate outliers [30]. RANSAC defines the optimal geometric model between two images by repeating random sampling of matched points.

**Figure 4:**
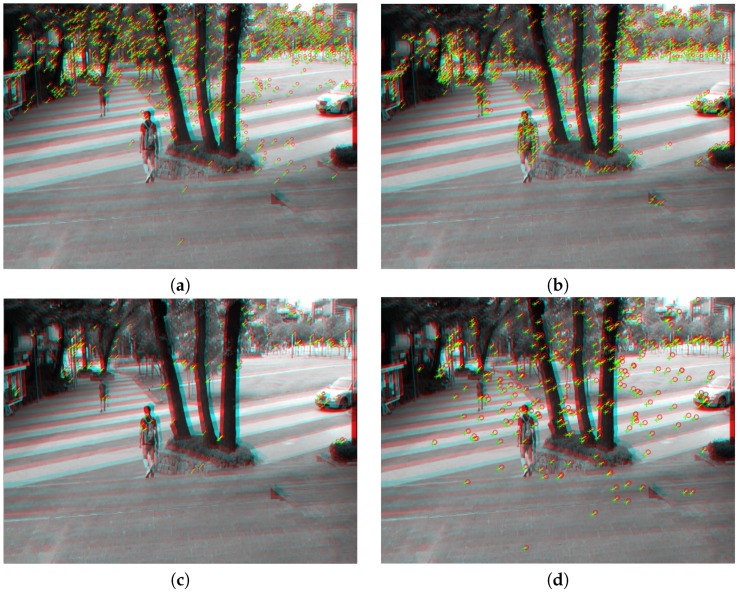
Experimental results of feature matching using: (**a**) SIFT, (**b**) SURF, (**c**) FAST and BRIEF, and (**d**) the proposed particle keypoint detection method.

Figure 4 shows feature detection results using the proposed method. Figure 4a shows matched points using SIFT with RANSAC, and Figure 4b shows matched points using SURF with RANSAC. Figure 4c shows matched points using FAST and BRIEF, and Figure 4d shows the results using the proposed particle keypoints. As a result, the particle keypoints can extract robust feature points of overall image including flat region.

## 4. Estimation of the Optimal Camera Path

Traditional video stabilization methods use a moving average of Gaussian filter to smooth the camera path. The moving average filter can smooth the camera path using the temporal mean of neighboring frames. The Gaussian kernel can remove undesired motion using the global transformation [7]. However, these methods fail to track a sharp change of the camera path. Furthermore, the performance of video stabilization becomes low when cropping regions and the amount of distortion increase. To solve this problem, the proposed method adaptively smooths the camera path using 1D TV algorithm [31]. The holes represent an empty region in a video frame which is generated after moving the frame by smoothed camera path. To compensate the holes, the boundary region of a stabilized video is generally cropped out, and the remaining central region is enlarged to fill the original size of the video frame. Therefore it is important to minimize the hole region to preserve the original contents. The stabilized video has less holes since the TV method can preserve the original path and removes undesired outliers.

Given the optimal homography $H_{t}$ between $f_{t-1}$ and $f_{t}$, a global camera path $C_{t}$ is generated. The corner points denoted as $V_{t}=\left\{\left(1,1\right),\left(1,h\right),\left(w,1\right),\left(w,h\right)\right\}$ in w×h input shaky frame $f_{t}$ are transformed to ${\hat{V}}_{t}$ by $H_{t}$. $H_{t}$ can be regarded as the transformation matrix of the camera movement. Therefore, the camera motion between $f_{t-1}$ and $f_{t}$ is simply considered as the difference between $V_{t}$ and ${\hat{V}}_{t}$. The global camera path $C_{t}$ is computed by adding the movement of adjacent frames as

(5) $$C_{t}=C_{t-1}+\left({\hat{V}}_{t}-V_{t}\right),$$

where ${\hat{V}}_{t}=H_{t}V_{t}$. The estimated global camera path $C_{t}$ is smoothed by 1D TV for video stabilization. The energy function for the smoothed camera path $P_{t}$ is defined as

(6) $$E\left(P_{t}\right)={∥P_{t}-C_{t}∥}_{2}^{2}+\lambda {∥AP_{t}∥}_{1},$$

where *A* the temporal difference matrix

$$A=\left[\begin{array}{ccccccc} -1 & 1 & & & & & \\ & & -1 & 1 & & & \\ & & & & \ddots & & \\ & & & & & -1 & 1 \end{array}\right],$$

and *λ* represents the weight coefficient for smoothing. The first term of Equation (6) enforces the smoothed camera path that is close to the original path, and the second removes noisy motions by smoothing the camera path. The energy function of Equation (6) can be minimized by the iterative clipping algorithm.

**Figure 5:**
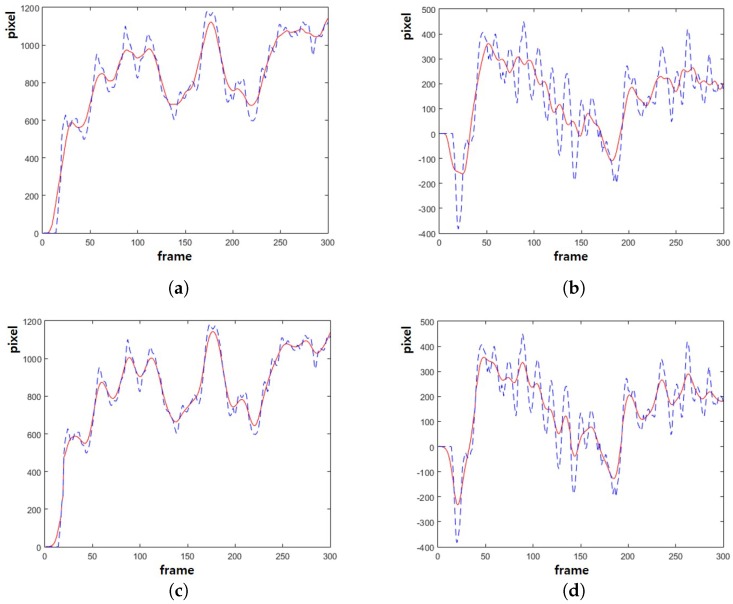
Results of camera path: (**a**) the *x*-coordinates of original camera path in dotted curve and the smoothed camera path using the moving average filter in solid curve, (**b**) the *y*-coordinates of original camera path in dotted curve and the smoothed camera path using the moving average filter in solid curve, (**c**) the *x*-coordinates of original camera path in dotted curve and the smoothed camera path using the proposed method in solid curve and (**d**) the *y*-coordinates of original camera path in dotted curve and the smoothed camera path using the proposed method in solid curve.

Figure 5 shows the estimated camera path using the proposed method. Figure 5a shows the *x*-coordinates of the original camera path in the dotted curve and the smoothed path using the moving average filter in the solid curve. Figure 5b shows the *y*-coordinates of the original camera path in the dotted curve and the smoothed path using the moving average filter in the solid curve. Figure 5c shows the *x*-coordinates of the original camera path in the dotted curve and the smoothed path using the proposed method in the solid curve. Figure 5d shows the *y*-coordinates of the original camera path in the dotted curve and the smoothed path using the proposed method in the solid curve. The proposed method can smooth the camera path without undesirable jitters and delay.

The final step of video stabilization is to reconstruct geometrically transformed frames using the smoothed camera path. The smoothed homography ${\hat{H}}_{t}$ can be estimated by the difference between the original camera path $C_{t}$ and the smoothed path $P_{t}$ as

(7) $$\left(C_{t}-P_{t}\right)+V_{t}={\hat{H}}_{t}V_{t},$$

where $V_{t}$ represents the four corner points of the image. The stabilized video frame ${\hat{f}}_{t}$ is generated by transforming using ${\hat{H}}_{t}$ as

(8) $${\hat{f}}_{t}={\hat{H}}_{t}f_{t}.$$

As a result, the proposed video stabilization method can successfully generate a stabilized video by estimating the optimal homography.

## 5. Experimental Results

This section presents experimental results and compares the performance of the proposed and existing methods. The proposed method improves the video quality by estimating the optimal homography using the particle keypoint update. To verify the accuracy of the estimated homography $H_{t}$ of temporally adjacent frames, $f_{t-1}$ and $f_{t}$, we tested the estimated projective transformation matrices from four feature different extraction methods, SIFT, SURF, FAST+BREIF, and the proposed method. We used SIFT and SURF algorithms with threshold values used in [24,25], respectively. Also, the proposed algorithm uses the intensity threshold t = 0.2 for FAST and a 256-bit string for BRIEF descriptor. After extracting feature points between $f_{t-1}$ and $f_{t}$, each transformation matrix is estimated. By combining all correspondences from the four methods, we evaluated the motion errors between the correspondences using *l*1-norm error evaluation as

(9) $$E_{1}=\frac{1}{n}\sum_{n}{∥{\tilde{X}}_{t}^{}-X_{t}^{}∥}_{1},$$

where ${\tilde{X}}_{t}=H_{t}X_{t-1}$ represents the transformed feature points in the previous frame $X_{t-1}=\left\{\left(x_{t-1}^{1},y_{t-1}^{1}\right),\cdots ,\left(x_{t-1}^{n},y_{t-1}^{n}\right)\right\}$, and $X_{t}=\left\{\left(x_{t}^{1},y_{t}^{1}\right),\cdots ,\left(x_{t}^{n},y_{t}^{n}\right)\right\}$ the feature points in the current frame. Table 1 summarizes the error of estimated homography using the four feature detection algorithms. The proposed method estimates the more accurate homography than other feature extraction methods as shown in Table 1.

**Figure 6:**
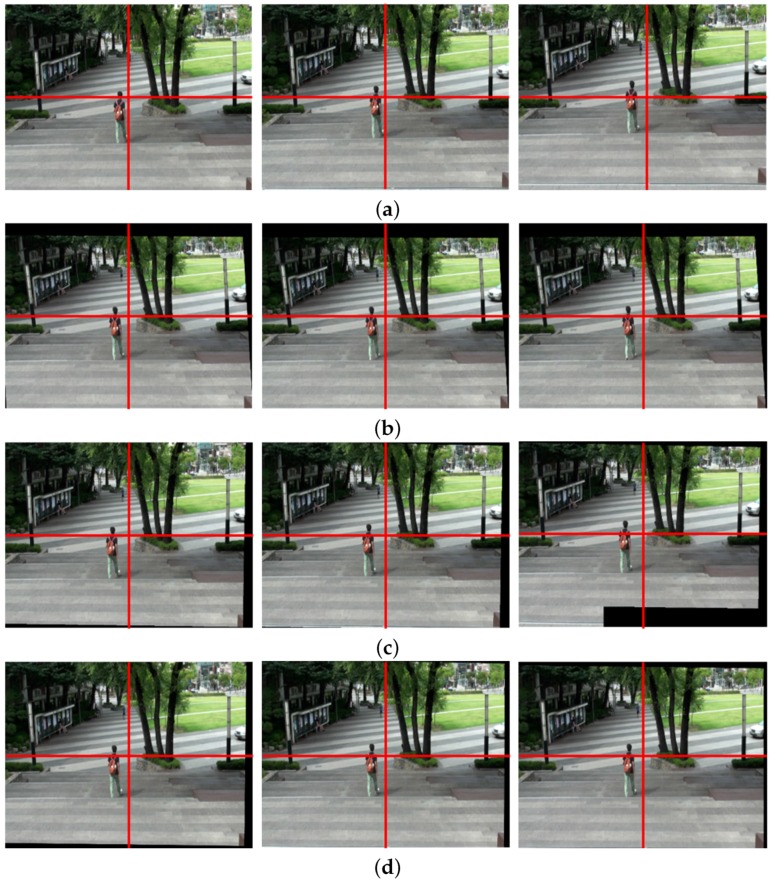
Experimental results of various video stabilization methods: (**a**) the input shaky video frames (80th, 81st, and 82nd frames), (**b**) the stabilized video using the global camera path using feature detection [7], (**c**) the bundled path algorithm [14], and (**d**) the proposed method.

Figure 6a shows the 80th, 81st, and 82nd frames in the original shaky video, and Figure 6b the correspondingly stabilized frames using the feature-based global camera path smoothing method [7], which cannot avoid a geometric distortion on the boundary because of the inaccurately estimated homography. We can easily find the distortion from the vertical structure on the right side of each frame. The bundled path algorithm fails in warping textureless blocks on the bottom of frame as shown in Figure 6c [14]. On the other hand, the proposed particle keypoint-based method can significantly enhance the shaky video with less geometric distortion on the boundary as shown in Figure 6d.

**Figure 7:**
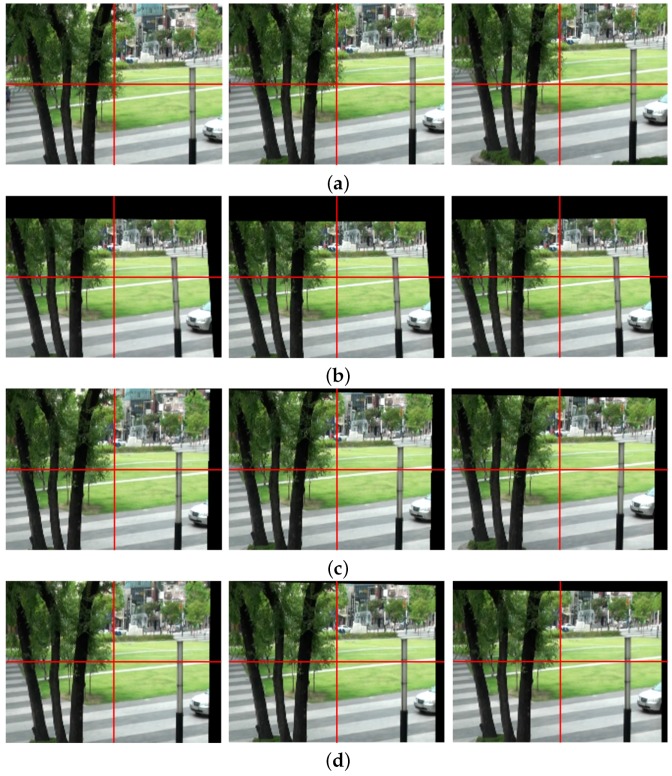
Experimental results of various video stabilization methods: (**a**) the expanded shaky video frames (80th, 81st, and 82nd frames), (**b**) the stabilized video using the global camera path using feature detection [7], (**c**) the bundled path algorithm [14], and (**d**) the proposed method.

Figure 7 shows the expanded version of an upper right region of Figure 6 for clearer comparison. The long object at the right side of each image is observed carefully. Figure 7a shows the expanded images of three temporally adjacent frames in original shaky video and Figure 7b shows the results of the stabilized video with geometric distortion by feature-based global smoothed camera path estimation method [7]. As shown in Figure 7c, the video stabilization method based on the bundled path could not successfully stabilize the video [14]. On the other hand, the proposed stabilized algorithm improves considerably the video quality with preserving the contents.

**Figure 8:**
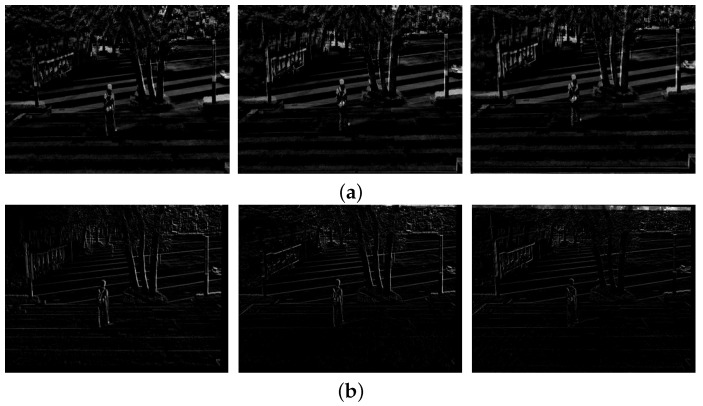
Experimental results of the video stabilization method: (**a**) differences of the original video (80th, 81st, and 82nd frames) and (**b**) the stabilized video (80th, 81st, and 82nd frames).

Figure 8 shows the difference of two temporally adjacent frames. Figure 8a shows the differences of three pairs of original frames {(79, 80), (80, 81), (81, 82)}. Figure 8b shows the differences of three pairs of stabilized frames {(79, 80), (80, 81), (81, 82)}. As shown in Figure 8, the proposed method can significantly compensate the undesirable movements.

**Figure 9:**
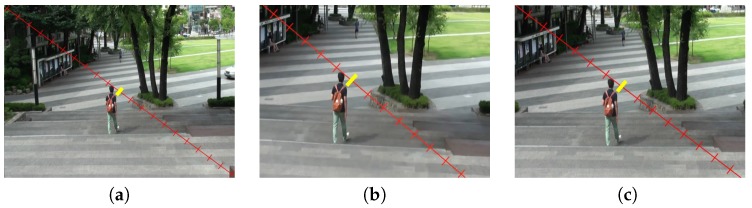
Experimental results of the video stabilization method: (**a**) the input shaky video frame (20/20), (**b**) the stabilized video using auto-directed (13/20) [32], and (**c**) the proposed video stabilization method (16/20).

To evaluate the empty region caused by the process of frame registration for stabilization, we compared the results of the proposed stabilization method and YouTube stabilizer using the same test video as shown in Figure 9. Stabilized frames are cropped to eliminate the missing boundaries, so it is important to have less cropping ratio to preserve the significant region of the original image. To measure the amount of cropping in various stabilization methods, tick marks are inserted on the diagonal line in the 80th input frame as shown in Figure 9a. Figure 9b,c respectively show the stabilized frames using auto-directed video stabilization method [32] and the proposed video stabilization method. As shown in Figure 9, the proposed video stabilization method can successfully preserve the contents of input frame with a reduced cropping ratio.

**Figure 10:**
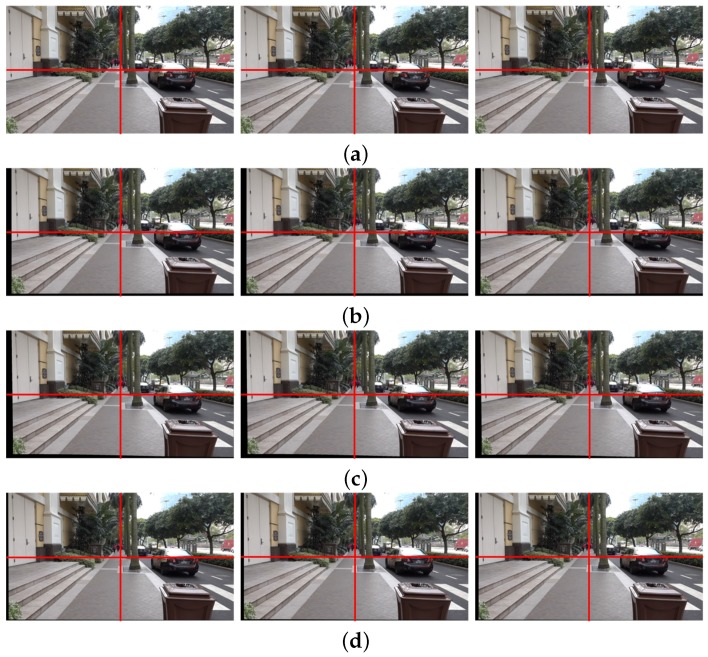
Experimental results of various video stabilization methods: (**a**) the input shaky video frames (170th, 171st, and 172nd frames), (**b**) the stabilized video using the global camera path using feature detection [7], (**c**) the bundled path algorithm [14], and (**d**) the proposed method.

Figure 10 shows the same test results of Figure 6 using different input video. Figure 10a shows the 170th, 171st, and 172nd frames of the input shaky video captured by a mobile camera. The significant portions of the stabilized video using the existing methods in [7,14] are removed by cropping to eliminate holes in the boundaries as shown in Figure 10b,c. As shown in Figure 10d, the stabilized video using the proposed method shows significantly improved video quality by removing undesired artifacts.

**Figure 11:**
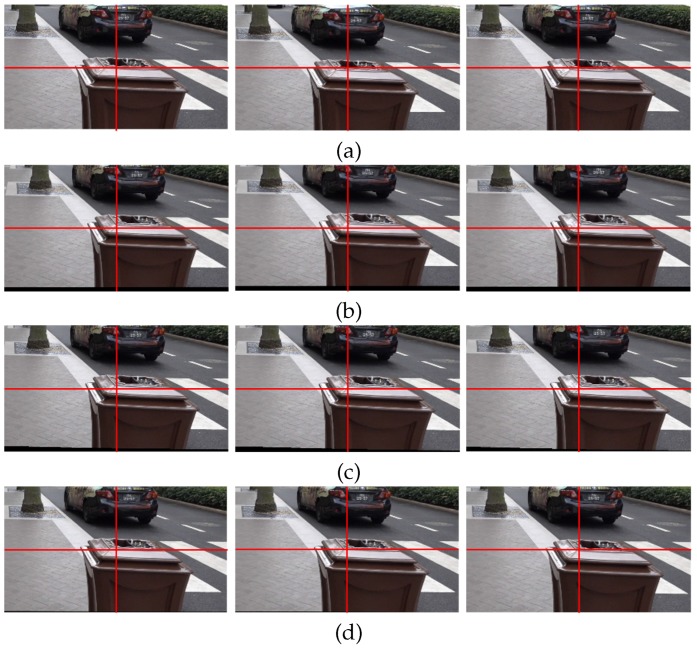
Experimental results of various video stabilization methods: (**a**) the enlarged shaky video frames (170th, 171st, and 172nd frames), (**b**) the stabilized video using the global camera path using feature detection [7], (**c**) the bundled path algorithm [14], and (**d**) the proposed method.

As shown Figure 11, a bottom right region of Figure 10 is enlarged to easily compare the results. Figure 11a shows the enlarged three original frames, and Figure 11b shows the stabilized results using the feature-based global camera path smoothing method [7]. Figure 11c shows the stabilized frames using the bundled path algorithm [14]. As shown in Figure 11d, the proposed method successfully obtains stabilized video with less holes.

**Figure 12:**
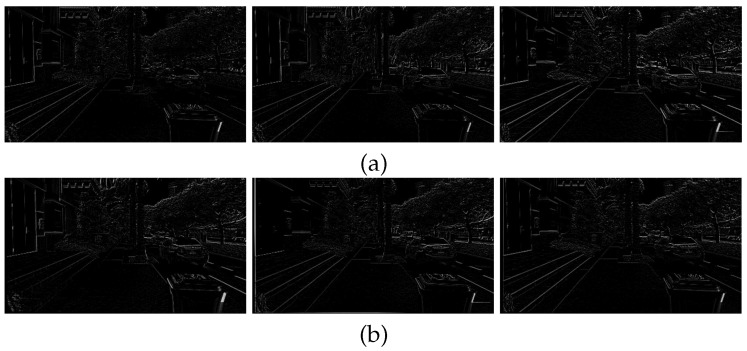
Experimental results of the video stabilization method: (**a**) differences of the original video (170th, 171st, and 172nd frames) and (**b**) the stabilized video (170th, 171st, and 172nd frames).

Figure 12 shows the same results of Figure 8 to demonstrate performance using the second test video. Figure 12a shows the differences of three pairs of original frames {(169, 170), (170, 171), (171, 172)}, and Figure 12b shows the differences of three pairs of stabilized frames {(169, 170), (170, 171), (171, 172)}.

**Figure 13:**
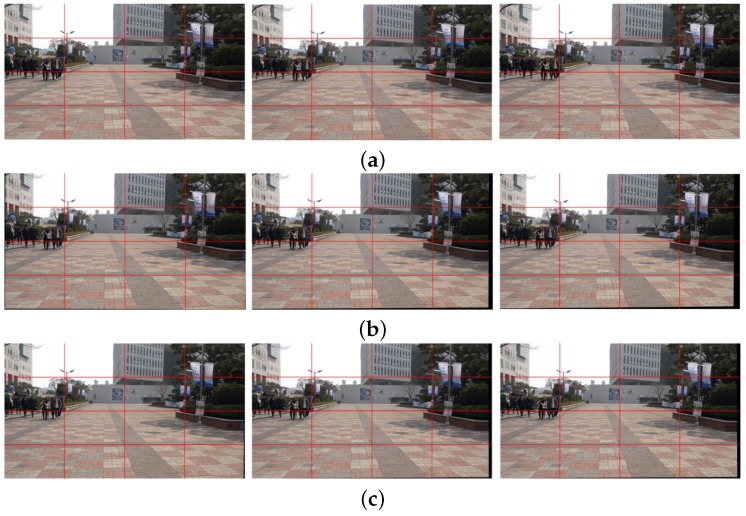
Experimental results of various camera path smoothing methods: (**a**) the input shaky video frames (275th, 276th, and 277th frames), (**b**) the stabilized video using a moving average filter [7], and (**c**) the proposed video stabilization method.

Figure 13 compares the performance of various camera path smoothing methods. Each resulting frame is divided into sixteen rectangular grids to easily evaluate the performance of the stabilization. Figure 13a shows the input shaky video frames acquired by a hand-held camera, and Figure 13b shows the results of stabilized video by smoothing the camera path using a moving average filter [7]. The stabilized frames using the proposed method that minimized the 1D TV are shown in Figure 13c. Based on comparing each grid, the proposed method can successfully enhance the shaky video with significantly reduced holes.

**Figure 14:**
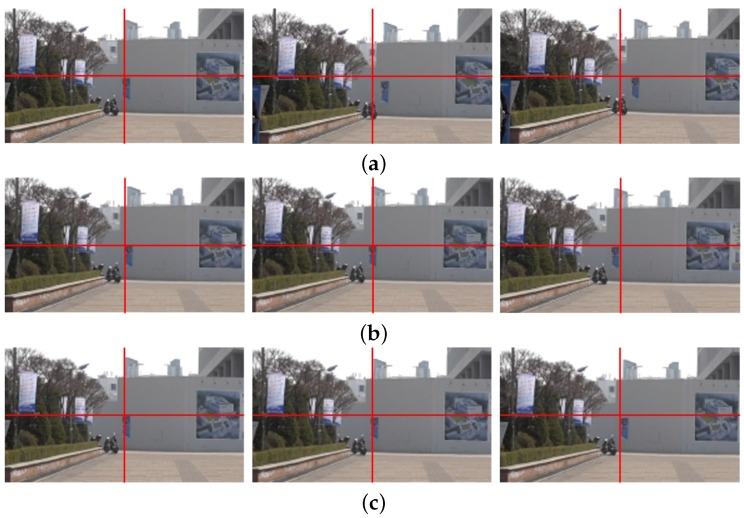
Experimental results of various camera path smoothing methods: (**a**) the enlarged shaky video frames (275th, 276th, and 277th frames), (**b**) the stabilized video using a moving average filter [7], and (**c**) the proposed video stabilization method.

Figure 14 shows the enlarged version of Figure 13. Figure 14a shows the first three frames in the original shaky video. Figure 14b shows the distorted object moving back and forth in the center of each frame. On the other hand, the proposed method successfully reduces the noisy motion of the shaky video as shown in Figure 14c.

**Figure 15:**
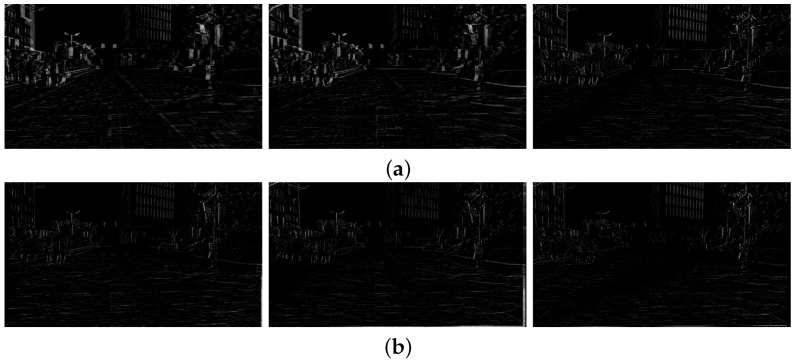
Experimental results of the video stabilization method: (**a**) differences of the original video (275th, 276th, and 277th frames), and (**b**) the stabilized video (275th, 276th, and 277th frames).

Figure 15 shows results of the difference of the successive two frames. Figure 15a shows the differences of three pairs of original frames {(274, 275), (275, 276), (276, 277)}. Figure 15b shows the differences of three pairs of stabilized frames {(274, 275), (275, 276), (276, 277)}.

The difference between two successive frames is minimized since the proposed method reduces the noisy motions. To evaluate the objective performance, we used the peak signal to noise ratio (PSNR) values of the temporally adjacent frames. The PSNR is defined as

(10) $$PSNR=10\log\frac{MAX_{f}^{2}}{MSE},$$

where $MSE=\frac{1}{M}\frac{1}{N}\sum_{x=0}^{M-1}\sum_{y=0}^{N-1}{∥f_{t-1}\left(x,y\right)-f_{t}\left(x,y\right)∥}^{2}$ represents the mean square error, and $MAX_{f}$ the maximum intensity value of the frames. Table 2 summarizes the PSNR values of adjacent video frames stabilized by the proposed method. As a result, the proposed video stabilization can correct the location of the pixels in the adjacent frames.

Finally, we measured the perspective distortion for objective assessment of the proposed video stabilization method using Liu’s method [14]. As mentioned in Section 2, a perspective distortion generally occurs when the real world is projected onto the image sensor. An inaccurately estimated homography results in the perspective distortion that significantly degrades the geometric quality of the video. For that reason, we estimated the perspective distortion using the transformation between the original and stabilized frames. The homography of the stabilized image sequences can be defined as

(11) $$P_{t}=B_{t}C_{t},$$

where $C_{t}$ and $P_{t}$ respectively represent the cumulative homographies between adjacent frames of the observed shaky and stabilized videos, and $B_{t}$ the transformation matrix. The perspective distortion is computed by averaging the perspective components in $B_{t}$ since the homography with distortion determines the video quality. Table 3, Table 4 and Table 5 summarize the perspective distortion of various video stabilization method. As shown in the Tables, the proposed video stabilization method can successfully remove the undesired motion without perspective distortion compared with conventional video stabilization algorithms.

**Figure 16:**
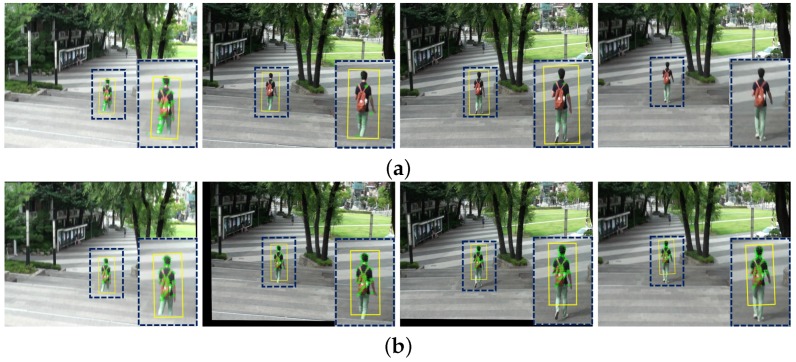
Experimental results of the object tracking: (**a**) shaky video and (**b**) stabilized video.

Unstable videos with undesired camera motions have the limited performance of object detection and tracking. The final experiment is performed to demonstrate whether the proposed method can play a practical role of pre-processing in various video analysis systems. We used the Lucas-Kanade feature tracker (LKT) to demonstrate the performance of the object tracking on shaky and stabilized videos. Figure 16 illustrates the experimental results of the object tracking. The yellow boxes in Figure 16 represent the tracking results using the LKT tracking method. Although the popular LKT algorithm tracked robust features with image rotation and view point change, it has a fundamental problem of missing the interest objects on the shaky video as shown in Figure 16a. As shown in Figure 16b, the proposed method can significantly improve the object tracking performance.

The stabilized results used in Figure 6, Figure 7, Figure 8, Figure 9, Figure 10, Figure 11, Figure 12, Figure 13, Figure 14, Figure 15 and Figure 16 using the proposed method can be found in the supplementary video with the comparison between the original and stabilized version.

## 6. Conclusions

The proposed video stabilization method removes unstable motions by estimating the optimal camera path using the robust keypoints extraction in the textureless region, and it smooths the shaky motions without frame delay using the variational optimization method. In addition, the proposed method is particularly suitable for hardware implementation in handheld cameras since it estimates the optimally camera path of shaky video using only four vertices in each frame. As a result, the proposed algorithm can successfully enhance the shaky video using an improved 2D stabilization method based on particle keypoints. The proposed method can be used for various video systems including mobile imaging devices, video surveillance systems, and vehicle imaging information systems. To overcome the vibration of the video acquired by vision-based mobile robots, the state of the art technology presents video stabilization system on a field programmable gate array (FPGA) based mobile robot system to apply to the single chip based embedded system for real-time video stream [33]. The proposed method can be applied to this system to extract correct features in the flat region and to improve the quality of stabilized video. Recently, an aerial surveillance system uses the video stabilization method to detect objects in a wide area [34]. The aerial video acquired with a moving camera cannot avoid jitters between temporally adjacent frames. For that reason, the video stabilization algorithm is an indispensable pre-processing step for robust detection of objects in the aerial surveillance system. The proposed method can define the significant feature points which is hard to be extracted in the flat or low-resolution region. It can significantly improve the performance of conventional video stabilization methods. The portable handheld camera users communicate with the dynamic activity videos such as walking, cycling, and hiking and it is important to remove undesirable shaky motion. The proposed feature extraction algorithm can be flexibly modified to extract robust initial keypoints, and it can also be used in a computationally powerful server-based cloud service to enhance the quality of the uploaded videos. The road videos in the first person can be stabilized by optimally estimating the camera path based on particle keypoints update in the flat region. Moreover, the personal videos nowadays are summarized in the form of the time lapse video because of the limited battery energy of the mobile devices and speed of the wireless network. In this context, the proposed method can be applied to the pre-processing step of a video summarization algorithm to remove wobble effects.

## Figures and Tables

**Table 1:** Error of the estimated homography using four different feature detection algorithms.

	Proposed Particle Keypoints	SIFT [24]	SURF [25]	FAST [26] + BRIEF [27]
video1	54.9254	56.3924	59.3830	60.6980
video2	11.9697	12.9337	13.1445	13.4249
video3	42.5910	43.3902	45.3725	44.0657

**Table 2:** Comparison of the original and stabilized videos in the sense of PSNR values.

	Frame	Original	Proposed
	80	15.9449	19.8664
	81	15.6589	21.3699
**video1**	82	15.7211	23.1769
	average (300 frames)	14.6864	17.4694
	170	19.7881	20.4265
	171	18.0865	19.7723
**video2**	172	16.4552	20.1274
	average (680 frames)	18.0661	20.0122
	275	17.2482	21.7773
	276	17.7494	21.8341
**video3**	277	20.0378	22.3599
	average (390 frames)	18.4174	19.4447

**Table 3:** Perspective distortion of the various video stabilization methods.

	Proposed	Single [7]	Bundled [14]
video1	0.000052	0.000068	0.000057

**Table 4:** Perspective distortion of the various video stabilization methods.

	Proposed	Single [7]	Bundled [14]
video2	0.000149	0.000178	0.000153

**Table 5:** Perspective distortion of the various video stabilization methods.

	Proposed	Moving average [7]
video3	0.0000925	0.0001070

## Supplementary Materials

The following are available online at http://www.mdpi.com/1424-8220/17/2/337/s1.
